# Prognostic value of isolated PR interval prolongation after transcatheter aortic valve implantation

**DOI:** 10.1093/europace/euag241

**Published:** 2026-09-03

**Authors:** Teodor Serban, Marc Salis, Arianna Bettelini, Céline Moser, Andrea Papa, Sven Knecht, Thomas Nestelberger, Christoph Kaiser, Gregor Leibundgut, Beat Schaer, Philipp Krisai, Felix Mahfoud, Michael Kühne, Christian Sticherling, Patrick Badertscher

**Affiliations:** Department of Cardiology, University Hospital Basel, Universitätsspital CH, Petersgraben 4, 4031 Basel, Switzerland; Cardiovascular Research Institute Basel, University Hospital Basel, Spitalstrasse 2, 4056 Basel, Switzerland; Department of Cardiology, University Hospital Basel, Universitätsspital CH, Petersgraben 4, 4031 Basel, Switzerland; Cardiovascular Research Institute Basel, University Hospital Basel, Spitalstrasse 2, 4056 Basel, Switzerland; Department of Cardiology, University Hospital Basel, Universitätsspital CH, Petersgraben 4, 4031 Basel, Switzerland; Cardiovascular Research Institute Basel, University Hospital Basel, Spitalstrasse 2, 4056 Basel, Switzerland; Department of Cardiology, University Hospital Basel, Universitätsspital CH, Petersgraben 4, 4031 Basel, Switzerland; Cardiovascular Research Institute Basel, University Hospital Basel, Spitalstrasse 2, 4056 Basel, Switzerland; Department of Cardiology, University Hospital Basel, Universitätsspital CH, Petersgraben 4, 4031 Basel, Switzerland; Cardiovascular Research Institute Basel, University Hospital Basel, Spitalstrasse 2, 4056 Basel, Switzerland; Department of Cardiology, University Hospital Basel, Universitätsspital CH, Petersgraben 4, 4031 Basel, Switzerland; Cardiovascular Research Institute Basel, University Hospital Basel, Spitalstrasse 2, 4056 Basel, Switzerland; Department of Cardiology, University Hospital Basel, Universitätsspital CH, Petersgraben 4, 4031 Basel, Switzerland; Cardiovascular Research Institute Basel, University Hospital Basel, Spitalstrasse 2, 4056 Basel, Switzerland; Department of Cardiology, University Hospital Basel, Universitätsspital CH, Petersgraben 4, 4031 Basel, Switzerland; Cardiovascular Research Institute Basel, University Hospital Basel, Spitalstrasse 2, 4056 Basel, Switzerland; Department of Cardiology, University Hospital Basel, Universitätsspital CH, Petersgraben 4, 4031 Basel, Switzerland; Cardiovascular Research Institute Basel, University Hospital Basel, Spitalstrasse 2, 4056 Basel, Switzerland; Department of Cardiology, University Hospital Basel, Universitätsspital CH, Petersgraben 4, 4031 Basel, Switzerland; Cardiovascular Research Institute Basel, University Hospital Basel, Spitalstrasse 2, 4056 Basel, Switzerland; Department of Cardiology, University Hospital Basel, Universitätsspital CH, Petersgraben 4, 4031 Basel, Switzerland; Cardiovascular Research Institute Basel, University Hospital Basel, Spitalstrasse 2, 4056 Basel, Switzerland; Department of Cardiology, University Hospital Basel, Universitätsspital CH, Petersgraben 4, 4031 Basel, Switzerland; Cardiovascular Research Institute Basel, University Hospital Basel, Spitalstrasse 2, 4056 Basel, Switzerland; Department of Cardiology, University Hospital Basel, Universitätsspital CH, Petersgraben 4, 4031 Basel, Switzerland; Cardiovascular Research Institute Basel, University Hospital Basel, Spitalstrasse 2, 4056 Basel, Switzerland; Department of Cardiology, University Hospital Basel, Universitätsspital CH, Petersgraben 4, 4031 Basel, Switzerland; Cardiovascular Research Institute Basel, University Hospital Basel, Spitalstrasse 2, 4056 Basel, Switzerland; Department of Cardiology, University Hospital Basel, Universitätsspital CH, Petersgraben 4, 4031 Basel, Switzerland; Cardiovascular Research Institute Basel, University Hospital Basel, Spitalstrasse 2, 4056 Basel, Switzerland

**Keywords:** TAVI, PR interval, Conduction disorders

Risk stratification for permanent pacemaker (PPM) implantation after transcatheter aortic valve implantation (TAVI) remains challenging.^1^ While several predictors have been identified, including baseline conduction abnormalities and QRS prolongation,^2–5^ the role of PR interval prolongation, particularly when occurring in isolation, has been insufficiently studied, despite its frequent occurrence after TAVI.^6,7^ The aim of the current study was to evaluate the association between isolated PR prolongation after TAVI and the risk of PPM implantation at 1 year.

We analysed a prospectively collected cohort of patients at the University Hospital of Basel, Switzerland, who underwent TAVI between September 2011 and March 2024. Adults (≥18 years) with available pre- and post-procedural 12-lead ECGs were included, while patients with prior pacemaker/ICD, redo-TAVI, non-sinus rhythm, missing data, intraprocedural complete AV block, or significant QRS prolongation (≥20 ms) were excluded. Both balloon-expandable and self-expandable valves were assessed.

Procedures followed standard imaging and implantation protocols, with patients monitored by continuous telemetry for 48–72 h. Serial ECGs were collected within 1 year before TAVI, within 24 h post-procedure, and then daily until discharge; the longest post-procedural PR interval was used to assess conduction changes. PR and QRS prolongations were defined as increases ≥20 ms, and isolated PR prolongation (study group) was defined as ΔPR ≥20 ms without ΔQRS ≥20 ms. Controls showed neither PR nor QRS prolongation.

The primary endpoint was PPM implantation within 1 year. Follow-up occurred at discharge, 30 days, and 12 months. Kaplan–Meier analyses and Cox proportional hazards models were used to assess the association between isolated PR prolongation and PPM implantation. Sensitivity analyses excluded pacemakers placed for indications other than high-degree AV block. To evaluate the prognostic relevance of the magnitude of PR prolongation, peri-procedural PR changes were calculated and analysed using multivariable regression adjusted for baseline or maximal PR interval, age, sex, QRS duration, and left ventricular ejection fraction (LVEF). A Bayesian hierarchical repeated-measures model using all serial ECGs provided complementary estimates of the association between PR dynamics and PPM implantation.

We included 603 patients [mean age 81.8 ± 6.4 years, 48% female, median Society of Thoracic Surgeons score 3% (IQR 2.0–4.9)], of whom 212 patients (35%) exhibited isolated PR prolongation, including 58 (27%) with a wide baseline QRS. The control group (*n* = 391) had no PR or QRS prolongation, with 86 patients (22%) presenting with a wide baseline QRS. Self-expandable valves were more frequently used in patients with isolated PR prolongation [163(77%) vs. 256 (66%), *P* = 0.005].

Following TAVI, the PR interval increased progressively. Among patients with isolated PR prolongation, 193 (91%) reached their maximum PR interval by Day 3; in the overall cohort, 546 (91%) reached their maximum by Day 4. Baseline and post-procedural QRS duration did not differ between groups. At 1 year, 54 patients (9%) underwent PPM implantation. The most common indication was high-degree AV block (40, 74%). Patients with isolated PR prolongation showed higher rates of PPM implantation compared with controls [28 (13%) vs. 26 (7%), *P* = 0.007, *Figure 1*]. One-year all-cause mortality did not differ between groups [13 (6%) vs. 29 (8%), *P* = 0.55].

**Figure 1:**
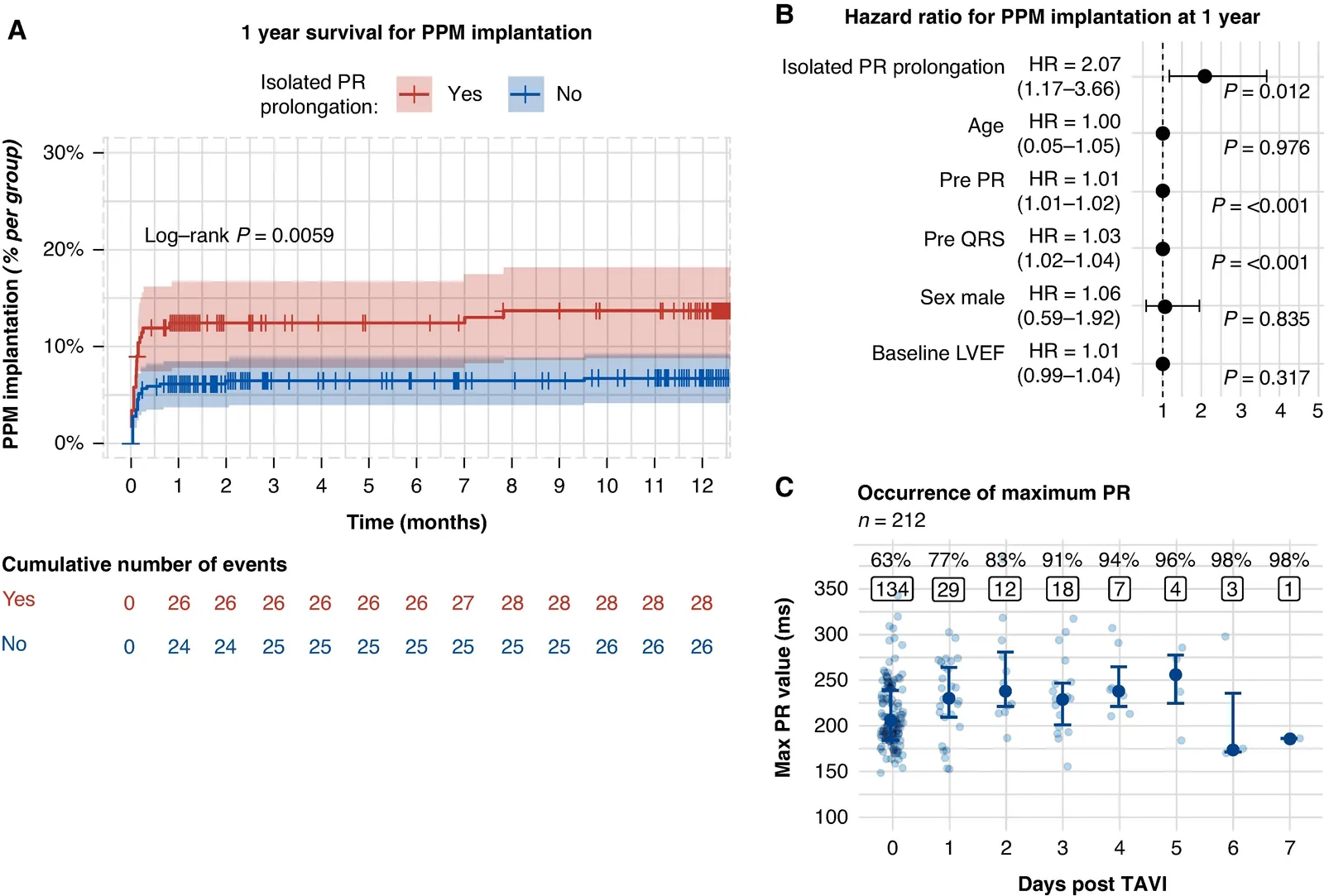
(*A*) One-year survival of PPM implantation stratified by presence of isolated PR prolongation. (*B*) Multivariable Cox regression analysis of isolated PR prolongation. (*C*) Day of maximum PR interval.

In multivariable Cox regression analysis, isolated PR prolongation was independently associated with PPM implantation at 1 year (aHR 2.07, 95% CI 1.17–3.66, *P* = 0.012, *Figure 1*), with consistent findings after excluding patients undergoing PPM implantation for indications other than high-degree AV block [22 (11%) vs. 18 (5%); aHR 2.3, 95% CI 1.2–4.41, *P* = 0.012]. The peri-procedural PR change was modestly larger in patients requiring PPM at 1 year but was not independently associated with PPM implantation after adjustment (aHR 0.92, 95% CI 0.83–1.03 per 10-ms increase, *P* = 0.14), a finding corroborated by the Bayesian model (Bayes aOR 0.81, 95% CrI 0.56–1.15). In contrast, the longest in-hospital PR interval remained independently associated with PPM implantation (aHR 1.18, 95% CI 1.09–1.28 per 10-ms increase; Bayes aOR 2.05, 95% CrI 1.44–2.97).

In this study, isolated PR interval prolongation was independently associated with an increased risk of PPM implantation within 1 year after TAVI, contrasting with previous findings^8^ and supporting the relevance of PR prolongation in current guideline-based post-TAVI risk stratification.^9,10^ Beyond the primary finding, several additional observations merit consideration.

First, the PR interval increased progressively after TAVI, with most patients reaching their maximum within 3 days. Second, baseline PR interval was independently associated with PPM implantation, consistent with prior studies. Importantly, however, the magnitude of PR change after TAVI was not independently associated with PPM implantation after accounting for the absolute PR interval reached. These findings suggest that the absolute post-TAVI PR interval may be more informative than the magnitude of PR prolongation alone. Thus, even modest PR prolongation may be relevant in patients with an already prolonged baseline PR interval, whereas a larger increase resulting in a relatively short absolute PR interval may carry less prognostic information.

This study has several limitations. First, it represents a retrospective analysis of a prospectively collected single-centre cohort, which may limit generalizability. Second, residual confounding cannot be excluded. Third, selection of the longest PR interval during hospitalization may have overestimated peak conduction delay. Furthermore, PPM implantation decisions were not standardized and may reflect physician discretion. However, the association persisted when the analysis was restricted to PPM implantation for high-degree AV block. These findings should therefore be considered hypothesis-generating and warrant confirmation in prospective studies.

In conclusion, isolated PR interval prolongation after TAVI was associated with an approximately two-fold higher risk of PPM implantation at 1 year. The absolute PR interval reached after TAVI, rather than the magnitude of PR change alone, may provide additional information for post-procedural risk stratification.

## Data Availability

The data underlying this article will be shared on reasonable request to the corresponding author.
